# Plants’ Response Mechanisms to Salinity Stress

**DOI:** 10.3390/plants12122253

**Published:** 2023-06-08

**Authors:** Thuvaraki Balasubramaniam, Guoxin Shen, Nardana Esmaeili, Hong Zhang

**Affiliations:** 1 Department of Biological Sciences, Texas Tech University, Lubbock, TX 79409, USA; thuvaraki.balasubramaniam@ttu.edu (T.B.); hong.zhang@ttu.edu (H.Z.); 2 Zhejiang Academy of Agricultural Sciences, Hangzhou 310021, China

**Keywords:** anthropogenic activities, ion homeostasis, salinization, salt tolerance, seed germination

## Abstract

Soil salinization is a severe abiotic stress that negatively affects plant growth and development, leading to physiological abnormalities and ultimately threatening global food security. The condition arises from excessive salt accumulation in the soil, primarily due to anthropogenic activities such as irrigation, improper land uses, and overfertilization. The presence of Na⁺, Cl^−^, and other related ions in the soil above normal levels can disrupt plant cellular functions and lead to alterations in essential metabolic processes such as seed germination and photosynthesis, causing severe damage to plant tissues and even plant death in the worst circumstances. To counteract the effects of salt stress, plants have developed various mechanisms, including modulating ion homeostasis, ion compartmentalization and export, and the biosynthesis of osmoprotectants. Recent advances in genomic and proteomic technologies have enabled the identification of genes and proteins involved in plant salt-tolerance mechanisms. This review provides a short overview of the impact of salinity stress on plants and the underlying mechanisms of salt-stress tolerance, particularly the functions of salt-stress-responsive genes associated with these mechanisms. This review aims at summarizing recent advances in our understanding of salt-stress tolerance mechanisms, providing the key background knowledge for improving crops’ salt tolerance, which could contribute to the yield and quality enhancement in major crops grown under saline conditions or in arid and semiarid regions of the world.

## 1. Soil Salinization: A Major Global Issue

Soil salinization is a global threat that affects 1100 Mha of soil, representing approximately 7% of the earth’s land surface [1]. As a multifactorial phenomenon, soil salinization occurs through natural geochemical processes and secondary anthropogenic activities [2]. Primary salinization events resulted from atmospheric deposition, elevation in the sea level, saltwater intrusion into freshwater aquifers, and rising temperature adversely have affected a significant part of the cultivated lands, whereas an estimated 30% of irrigated lands are aggravated by secondary salinization due to excessive fertilizer usage, poorly managed practices, and intensified agriculture [3,4]. The scarcity of fresh water for irrigation and the ongoing degradation of agricultural lands attributable to salt stress causes substantial losses in agricultural productivity, particularly in arid and semiarid areas. Soil salinization began over hundreds to thousands of years ago. It has now degraded areas such as the Aral Sea Basin in Central Asia, the Yellow River Basin in China, the Murray-Darling Basin in Australia, and the San Joaquin Valley in California at an alarming pace in the last 50 years [5]. 

The occurrence of soil salinization is mainly due to the accumulation of water-soluble salts, including sodium (Na^+^), potassium (K^+^), chloride (Cl^−^), and sulfate (SO_4_^2−^) in the root zone, which causes osmotic variations, reducing the ability of plant root cells to absorb water from soil [6,7,8]. The existence of the salt ions results in hyperionic salt stress, which is detrimental to plant cells. Among the salinity-causing water-soluble salts, Na^+^ and Cl^−^ are considered the major ions contributing to soil salinity, and the excessive Na^+^ among the exchangeable cations contributes to sodicity [9]. Ancient cells were built to survive salinity since the early evolution of life originated in primeval oceans with similar or even more salt than contemporary oceans [10]. Hence, many terrestrial plants can tolerate low to moderate salinity, whereas naturally occurring salt-tolerant plants, or halophytes, may thrive at high salinity levels. Most crop species today fall into the glycophyte category and cannot grow well in saline environments. In fact, many crops such as tomatoes and rice are very sensitive to soil salinity. The deleterious impacts of salinity on plants are manifested first with a short-term osmotic stress, then with the long-term accumulation of phytotoxic ions [11]. The salt-induced osmotic stress occurs at the initial stage of exposure to salt due to the incremental uptake of salts and subsequent reduction of water potential around the root zone, which diminishes water conductivity in plant cells and primarily leads to hindered plant growth [12,13]. Prolonged exposure to high salinity causes the accumulation of toxic ions such as Na^+^, Cl^−^, and SO_4_^2−^, which induce ion toxicity and impair nutrient uptake, exacerbating the damage to plant cells and tissues [14]. The undesirable consequences of salt stress on plants are manifested in morphology (stunted growth, chlorosis, and impaired seed germination), physiology (inhibition of photosynthesis and nutrient imbalance), and biochemical properties (oxidative stress, electrolyte leakage, and membrane disorganization) [15,16]. The adverse effects of salinity are particularly profound at the reproductive stage [17]. A plethora of research has been conducted and reviewed on salt-induced damages and their impacts on plants over the last 20 years.

An increased tolerance to salt stress is one of the major emphases in the genetic improvement of crops of agricultural, environmental, horticultural, and economic importance. Thus far, conventional breeding methods and innovative genetic engineering approaches have achieved significant advances in obtaining more salt-tolerant plants. Studying salt tolerance is a critical component of plant biology research, enabling us to understand the complicated mechanisms of salt tolerance in plants and explore strategies to ameliorate the detrimental effects of salt stress. This review summarizes the most recent research activities in plant response to salt stress and the potential salt tolerance mechanisms, which could guide future efforts in creating highly salt-tolerant crops. 

## 2. Impacts and Consequences of Salt Stress on Plants: A Challenge for Sustainable Agriculture

### 2.1. Growth of Plants

Like many other abiotic stresses, salt stress suppresses plant growth, and the rate of growth reduction depends on several factors, such as plant species, developmental stage, and the concentration of salt [18]. Stunted growth is an adaptive mechanism for survival, which allows plants to combat salt stress [19]. Salt stress might reduce the expression of key regulatory genes involved in cell cycle progression (e.g., cyclin and cyclin-dependent kinase), leading to decreased cell numbers in the meristem and a growth inhibition which impacts the plant’s ability to absorb nutrients and water efficiently. Certain plants “panic” immediately upon salt-stress exposure, react quickly, and stop growing. In contrast, others do not respond adequately and face the danger of dying by continuing to grow under severe salt-stress conditions [20]. 

The plant cell shrinks and dehydrates immediately after salt stress is imposed; however, it is recovered later. Despite this recovery, cell elongation, and to a lesser extent, cell division, is affected, resulting in a lower root and leaf growth rate. After the occurrence of salinity stress, the lateral shoot enlargement is affected, leading to apparent differences in overall growth and injury between salt-stressed plants and their nonstressed controls. This response is due to changes in the cell–water relation resulting from osmotic changes outside the root (osmotic effect). The osmotic effect leads to a reduction in the capability of plants to absorb water. The effects of salinity on plant growth have been extensively studied in various plant species, including *Eruca sativa* Mill [21] and *Fragaria × ananassa* Duch [22]. 

### 2.2. Photosynthesis

Photosynthesis is plants’ most widely recognized and vital characteristic, which converts solar energy into chemical energy. Various factors, including impaired chlorophyll biosynthesis [23], altered enzymatic activity [24], stomatal closure, reduced CO_2_ supply [25], and damaged photosynthetic apparatus [26], are correlated with a salt-induced photosynthetic reduction. The decline in chlorophyll content has been reported under salt-stress conditions due to increased oxidation and degradation of chlorophyll initiated by the accumulation of reactive oxygen species (ROS), and the chlorophyll reduction is proportional to the level of salinity [27,28]. Pseudocyclic electron transport resulting from inhibiting the electron transport chain causes an excessive production of ROS [29]. Consequently, ROS alters photosynthetic proteins and the photosystem assembly [30]. In addition, exposure to short-term salt stress at higher concentrations disturbs the dynamics of the chloroplast ultrastructure by inducing thylakoid swelling and starch accumulation [31]. Plenty of studies have been performed on various plant species, including *Solanum melongena*, *Portulaca oleracea*, *Oryza sativa*, and *Jatropha curcas*, on the impacts of salts on photosynthesis, and all these studies reached the same conclusion that salt stress leads to reduced photosynthetic rates at low salt concentrations and severely damages chloroplast structures and photosynthetic machinery at moderate to high salt concentrations [16,32,33,34].

### 2.3. Nutrient Balance 

The growth of plants in the absence of salinity is typically represented by the “generalized dose–response curve” [35] in relation to the concentrations of essential nutrient elements in the root media. Plant growth under suboptimal levels of nutrients around the root zone may be impeded due to either a nutrient-induced deficiency or toxicity. The difficulty of mineral nutrient acquisition under salt-stress conditions can be attributed prevalently to the reduction of nutrient availability due to the competition with major ions (Na^+^ and Cl^−^). Such interaction frequently results in deficiencies in Ca^2+^, K^+^, and Mg^2+^ [36]. The relationship between salt stress and essential mineral nutrients such as nitrogen, phosphorous, and potassium is complex. Nitrogen is an essential mineral element and constituent of plant cellular components. Under saline conditions, an increased uptake and accumulation of Cl^−^ can decrease the total shoot nitrogen uptake due to Cl^−^/NO_3_^−^ antagonism [37]. Salt stress also affects the uptake of phosphorous, which is required for photosynthesis, storage, and energy transfer. When the soil contains excessive Cl^−^ and SO_4_^2−^, phosphorous uptake gets reduced, possibly due to the high ionic strength of the media and low solubility of the Ca ± P minerals. Potassium is a vital inorganic solute necessary for protein synthesis and water relations. Under saline conditions, there is intense competition between K^+^ and Na^+^. The cellular balance between sodium and potassium is essential for plant survival in saline soil. However, they both have a molecular similarity, which causes potassium replacement by sodium even though it cannot take over the function of potassium in cellular processes. An increased Na^+^ concentration decreases K^+^ and Ca^2+^ concentrations, as Na^+^ and K^+^ compete at root uptake sites. The reduction in K^+^ uptake in plants caused by Na^+^ is a competitive process, regardless of whether the solution is dominated by Na^+^ salts, Cl^−^, or SO_4_^2−^ salts [36]. A substantial body of information in the literature, including *Manihot esculenta* and *Zea mays*, indicates that salinity causes nutrient imbalances and reduces crop productivity [38,39,40,41,42].

### 2.4. Water Relations

The rapid absorption of ions leads to the accumulation of ions in plant cells, negatively affecting plant–water relations. Under salinity stress, the osmotic potential of plant cells becomes more negative due to the presence of a high salt concentration in the soil, which creates an osmotic gradient that drives water out of the plant cells and decreases turgor pressure [43]. Research with *Corchorus olitorius* by Chaudhuri and Choudhuri (1997) showed a decrease in various parameters, such as relative water content, water uptake, and transpiration rate, when plants are exposed to short-term salinity stress [44]. Recent studies further affirm the abovementioned results [45,46]. The osmotic potential in the rooting medium and the mode of imposed salinity stress determines the magnitude of plant cells’ decline in leaf water potential and osmotic potential. Maintaining turgor pressure at the steady-state level is achieved in plants by reducing their osmotic potential compared to the total water potential under progressive salinity stress [47]. Pertaining to the water movement, in general, water moves from the soil to the root xylem via an apoplastic path driven by a hydrostatic pressure gradient under transpiring conditions. However, when transpiration is limited by salinity, water flows across membranes mainly through the cell-to-cell path [46,48,49,50]. 

### 2.5. Yield

Apart from the plant developmental aspects discussed above, salinity also impairs protein synthesis, energy metabolism, and cell signaling. Therefore, it ultimately hinders agricultural productivity by necessitating a high metabolic expenditure for plant adaptation, growth maintenance, and stress responses, which causes an overall decrease in yield [51]. Salt-induced osmotic stress and salt absorption rate determine the biomass yield reduction and intensity of the subsequent membrane injury, respectively [52]. The concept of defining a threshold level of salinity at which yield is reduced drastically was introduced by Maas Hoffman in 1977 [53]. Thus far, various studies have been conducted in various plant species including bioenergy grass *Miscanthus × giganteus* and *Sorghum bicolor* to investigate crop yield responses under salinity conditions [54,55,56]. A comprehensive understanding of the pervading consequences of salinity stress on plants could assist scientists in fine-tuning the salt-induced response and eventually leading to the improvement of crop productivity under salinity stress.

## 3. Alleviation of Salt Stress by Various Strategies in Plants

Plants have evolved flexible systems to cope with salinity stress by changing at the morphological, physiological, biochemical, and molecular levels. Salt tolerance can be attained by managing cytoplasmic ion content, which involves ion homeostasis and compartmentalization, osmotic adaptation, and increased antioxidation metabolism, such as a higher capacity for scavenging ROS [57,58]. Several endogenous phytohormones such as abscisic acid (ABA), auxin, salicylic acid (SA), jasmonic acid (JA), cytokinins, gibberellins, ethylene, and brassinosteroids (BR) are critical in modulating plant response to salinity stress, and in the establishment of a higher salinity tolerance [59,60]. Many salinity-stress-specific genes and transcription factors are upregulated upon exposure to salinity stress, enabling plants to adapt to the salt-stress environment.

### 3.1. Accumulation of Osmotic Adjustment Substances

The accumulation of compatible organic solutes such as proline, soluble sugars, glycine betaine, and polyols is one of the most common responses in plants grown under salt-stress conditions. These soluble and low molecular weight compounds serve as osmoprotectants and contribute to the intracellular osmotic adjustment and ROS detoxification while protecting the membrane structure without impairing cellular metabolism [61].

Glycine betaine is a quaternary ammonium compound that accumulates abundantly in response to dehydration and salt stress in many plants [62,63]. Glycine betaine is synthesized in the chloroplast, where it accumulates to play a role in the osmotic adjustment of the thylakoid membrane, therefore maintaining photosynthetic efficiency [64]. In response to salinity stress, glycine betaine is synthesized in many plants to alleviate the adverse effects of salt stress to maintain the osmotic status of the cell. Glycine betaine is studied broadly by modifying its metabolic pathways through transgenic approaches. For example, the betaine aldehyde dehydrogenase gene from the halophyte plant *Suaeda liaotungensis*, which encodes an enzyme converting the betaine aldehyde to betaine, is overexpressed in transgenic tobacco plants and has exhibited a significantly increased salt tolerance [65]. The choline oxidase gene from *Arthrobacter globiformis* was introduced into the Indica rice, and the transgenic rice could tolerate salt stress up to 150 mM. The elevated salt tolerance is possibly due to the conversion and consequent accumulation of glycine betaine from choline catalyzed by choline oxidase as a two-step oxidation reaction [66]. Apart from the success of engineered plants in increasing salt tolerance, other techniques were also used. For example, exogenous application of glycine betaine in common beans (*Phaseolus vulgaris* L.) substantially reduced the uptake of Na^+^, induced the uptake of K^+^ and therefore maintained an elevated ratio of K^+^/Na^+^, which enhanced the salt tolerance in common beans [67]. Another group of scientists illustrated that the exogenous application of glycine betaine in *Dalbergia odorifera* enhanced plant growth [68].

Proline, as a vital compatible osmolyte and antioxidant, rises under salinity stress and it assists plants in maintaining cell turgor. The elevated amount of proline content has been identified and used as a physiological hallmark in plant response to salinity stress [69]. The expression of genes involved in proline biosynthesis is activated by salinity stress, which subsequently leads to the production and accumulation of proline in plant cells [70,71]. Pyrroline-5 carboxylate synthetase (P5CS) is an enzyme responsible for catalyzing the first step in proline biosynthesis. P5CS1 is one of the two isoforms of P5CS and plays a vital role in salt-stress-induced proline accumulation. Studies have shown that knocking out *P5CS1* in *Arabidopsis thaliana* leads to a hypersensitivity to salt stress [72]. An exogenous application of proline in *Capsicum annuum* L. significantly reduced the inhibitory effects of salinity and improved plant growth and yield under salt stress conditions [73]. Proline accumulation in the desert plant *Pancratium maritimum* L. under salinity conditions helps maintaining the activities of antioxidative enzymes, upregulating the production of stress-protective dehydrin proteins and improving salt tolerance [74].

Soluble sugars predominantly comprise glucose, sucrose, and trehalose, and they are involved in the salt stress tolerance of plants. A higher accumulation of soluble sugars protects soluble enzymes from the toxicity of greater concentrations of intracellular inorganic ions under salt stress [75]. For example, a distinctive feature of trehalose that enables reversible water absorption capacity could protect molecules from osmotic damage [76]. Transgenic Arabidopsis plants overexpressing a salt-related wheat gene *TaSST* exhibited more soluble sugar content than wild-type Arabidopsis plants under a NaCl treatment [77]. Despite the involvement of soluble sugars, polyols also serve as compatible solutes and scavengers of ROS. For example, mannitol, an acyclic polyol, plays a substantial role in osmotic regulation and enhances salt tolerance in higher plants. Pujni et al. (2007) introduced the *E. coli* mannitol-1-phosphate dehydrogenase gene (*mt*/*D*), a gene involved in mannitol synthesis, into Indica rice, and the transgenic rice plants exhibited a correlation between the increased salt tolerance and the mannitol accumulation [78]. Therefore, the knowledge of the relevant mechanisms of specific osmolytes could be helpful in the generation of salt-tolerant crops.

### 3.2. Ion Homeostasis and Compartmentalization

The ionic imbalance caused by salinity stress affects many aspects of plant growth and development. For example, cellular metabolism, photosynthesis, and root architecture are interrupted by a reduced uptake of mineral nutrients under salt-stress conditions. However, plants have evolved to maintain intracellular ion homeostasis by controlling ion influx and its compartmentalization to cope with salinity stress. Ion homeostasis refers to a basic dynamic process in plants involving an energetically costly gradient to uptake required ions and eliminate toxic ions. Plants, whether glycophytes or halophytes, are vulnerable to greater concentrations of Na^+^ in the cytoplasm. Sodium uptake in plants occurs predominantly at the root–soil interface, and it is likely facilitated by nonselective cation channels, such as cyclic nucleotide-gated channels (CNGCs) and glutamate receptors (GLRs). In addition, high-affinity potassium transporters (HKTs) and aquaporins are also employed in Na^+^ uptake in plants [79,80]. The translocation of Na^+^ from roots to shoots occurs through the apoplastic pathway, transitioning to the symplast of the root epidermis before being loaded into the tracheids of the xylem, eventually reaching the shoots, particularly the leaf blades, where its effects are most pronounced [6]. Overall, plants have developed different strategies to protect the cytoplasm against the toxic effects of Na^+^ by restricting the Na^+^ influx into the cell, enhancing the Na^+^ exclusion out of the cell, and maximizing the compartmentalization of Na^+^ into the vacuole.

#### 3.2.1. Salt Overly Sensitive (SOS) Genes

Of the three main strategies, exporting Na^+^ to an external medium or apoplast has been demonstrated by the well-defined salt overly sensitive (SOS) signaling pathway that involves three genes, *SOS1*, *SOS2*, and *SOS3*, in *Arabidopsis thaliana* [81,82,83]. Among the three loci, *SOS1* controls the ion homeostasis of essential ions such as K^+^ and Ca^2+^ and the subsequent achievement of salt tolerance. The *SOS1* gene encodes a plasma membrane-localized Na^+^/H^+^ antiporter that exports a Na^+^ in exchange for a proton [81]. The necessity of SOS1 for controlling the long-distance movement of Na^+^, the capacity of Na^+^ efflux, and the successive maintenance of low concentrations of Na^+^ in root cells was demonstrated by physiological, genetic, and biochemical analyses [81]. The *SOS1* complementation of yeast Na^+^/H^+^ antiporter mutants and the preferential expression of *SOS1* in parenchyma cells at the xylem/symplast boundary has indicated that SOS1 is involved in ion efflux from the cytosol to the surrounding medium and regulating Na^+^ retrieval from xylem sap [84]. Several studies have shown that either an overexpression or co-overexpression of *SOS1* with other salt-tolerant genes leads to a significantly enhanced salt stress tolerance in various plants, including Arabidopsis [84,85,86], tobacco [87], and rice [88].

#### 3.2.2. High-Affinity Potassium Transporters (HKTs)

HKT transporters are usually known as monovalent cation transporters and belong to the Trk/Ktr/HKT superfamily. They have been broadly characterized in various plants and have been shown to play a critical role in salt tolerance by excluding Na^+^ ions from sensitive shoot tissues of the plants [89,90,91]. On their transport selectivity, HKTs can mediate Na^+^ import and thereby can be considered Na^+^/K^+^ symporter, and some members of HKTs may mediate Mg^2+^/Ca^2+^ permeability across the plasma membrane of plant cells [92]. The structural feature of HKTs is defined by four repetitions of MPM, where “M” refers to the transmembrane segment and “P” refers to the “pore-loop domain”. The assembly of the repetition M1_A_-P_A_-M2_A_–M1_D_-P_D_-M2_D_ and the structural determinant located in the first P domain, P_A_, define the two categories of HKTs: HKT1-type and HKT-2 type [89]. HKT2-type proteins carry a Gly residue at the MP_A_M motif that determines the permeability of Na^+^ or K^+^, therefore acting as a Na^+^/K^+^ symporter. When Gly is substituted by Ser, HKT transporters solely exhibit a Na^+^ selective permeability and fall into the HKT1-type category [93], where they inhibit the transport and accumulation of Na^+^ in shoots by unloading excessive sodium from xylem sap and sequester it into xylem parenchyma cells [94].

#### 3.2.3. Proton Pumps

Both glycophyte and halophyte plants maintain cytosolic Na^+^ concentration at nontoxic levels by compartmenting the excessive Na^+^ in the vacuole or exporting them out of the cytosol, which are considered key mechanisms in averting the deleterious effects of salinity. Regulating toxic levels of Na^+^ accumulation and escalating K^+^ uptake are crucial events in salt tolerance, and they are broadly interpreted in the context of a high cytosolic K^+^/Na^+^ ratio. Consequently, membrane proton pumps, ion transporters, and channels become part of salt-stress-tolerance mechanisms in plants. The primary plant proton pumps, alias “work horses”, include plasma membrane H^+^-ATPase (P-type H^+^ ATPase), vacuolar H^+^-ATPase, and vacuolar H^+^-pyrophosphatase (H^+^-PPase). P-type H^+^ ATPase is composed of a single polypeptide and embedded in the plasma membrane as a homodimer molecule. Although all plant tissues contain several isoforms of P-type H^+^ ATPase to varying degrees, some isoforms exhibit a spatial expression more specifically, as indicated by Sussman in 1994 [95]. Apart from the salt-tolerance trait of energizing the secondary transporters, P-type H^+^ ATPase also has housekeeping functions such as mediating the turgor pressure, cell wall extension, and intracellular pH. Meanwhile, vacuolar H^+^-ATPase is a complex, bipartite structure assembled as two major subcomplexes: an integral membrane V0 complex and a peripheral V1 complex representing the proton transporter and the driven ATPase. The significance of this proton pump is well characterized under salt-stress conditions and thus has been portrayed as an ecoenzyme. In contrast with the vacuolar H^+^-ATPase, the vacuolar H^+^-pyrophosphatase is a single polypeptide composed of a homodimer with subunits of 80 kDa. This electrogenic proton pump utilizes inorganic pyrophosphate (Mg_2_PP_i_) as the energy source to generate a proton gradient difference for the uphill transport of protons from the cytosol to the vacuolar lumen. The membrane-bound H^+^-PPases were encoded by three genes in *Arabidopsis thaliana*. Of those, only one H^+^-PPase, also known as type-1 H^+^-PPase (AtVHP1 or AVP1), is targeted at the vacuolar membrane [96]. Vacuolar H^+^-PPases are not merely functioning in regulating cytosolic PPi homeostasis but also generate an electrochemical proton gradient across the vacuolar membrane, which leads to a proton motive force (PMF). Under salinity-stress conditions, the PMF is utilized by secondary transporters such as proton-coupled Na^+^ and K^+^ antiporters to actively sequester excessive toxic ions from the cytosol into the vacuole [97,98]. A vast exploration of *AVP1*’s function has been conducted by overexpressing *AVP1* alone or co-overexpressing with other beneficial genes to study salt tolerance in Arabidopsis [99,100,101,102,103], creeping bentgrass [104], cotton [105,106,107,108], peanut [109], and barley [110].

#### 3.2.4. Na^+^/H^+^ Antiporter (NHX)

Several transporters function in counteracting the accumulation of Na^+^ and eliminating its noxious effect in the cytosol. The vacuolar NHXs sequester a Na^+^ into the vacuole in exchange with a H^+^. These monovalent ion exchangers in Arabidopsis are categorized into the NHX family with eight members based on their subcellular localization. AtNHX7/SOS1 and AtNHX8 are localized to the plasma membrane, and AtNHX1-4 are localized to the vacuolar membrane, while the rest of the members AtNHX5 and AtNHX6 are localized to the inner membrane (AtNHX5 on the Golgi membrane also assists in trafficking Na^+^ into the vacuole) [111]. An elevated expression of *AtNHX1* leads to an increased salt tolerance in various plant species such as Arabidopsis [85,112], rice [113,114], cotton [115,116], and tomato [117].

Plants also minimize salt injury by restricting Na^+^ influx to the aerial parts, mainly actively growing and photosynthesizing shoots and leaves. Apoplastic barriers and Na^+^ immobilization are considered effective strategies for reducing the accumulation of Na^+^. Casparian strips and suberin lamellae function as extracellular hydrophobic apoplastic barriers located in the endodermal cell wall and play important roles in restricting the free diffusion of solutes. A Casparian strip in the endodermal root cells imposes a restriction barrier to the movement of ions from root to shoot. Ions must move from the apoplast to the symplast pathway to cross the cell membrane and move through the endodermis to avoid the Casparian barrier. This transition pathway allows plants to partially exclude harmful ions, thus restricting their movement to the xylem. Suberin lamellae play a relatively minor role by reducing Na^+^ leakage into endodermal cells, reducing plant energy requirements. Several studies have reported reinforcing apoplastic barriers as an effective approach for reducing Na^+^ influx. For instance, Krishnamurthy et al. (2011) demonstrated that extensive apoplastic barriers in roots led to a reduced Na^+^ uptake, enhanced survival, and salt-stress tolerance in *Oryza sativa* L. [118].

The above-mentioned transporters and channels related to salt-stress tolerance contribute to the uptake, transportation, and distribution of Na^+^ and K^+^. Several other important transporters, such as K^+^ uptake permease (KUP)/K^+^ transporter, stelar K^+^ outward rectifier (SKOR), and Ca^2+^/cation exchanger have been cloned and shown to maintain cytosolic ion homeostasis under salt-stress conditions.

### 3.3. Oxidative Stress and Antioxidant Defense under Salt-Stress Conditions

The salt-induced accumulation of ROS has a strong oxidative ability, which causes oxidative damage to membrane lipids, proteins, and nucleic acids, causing irreversible metabolic dysfunction. Plants have antioxidant enzymes and nonenzymatic molecules to detoxify salt-induced ROS that are probably generated from the electron transport chains of mitochondria and chloroplasts. Superoxide dismutase (SOD), catalase (CAT), peroxidase (POX), and ascorbate peroxidase (APX) are the antioxidant enzymes, whereas glutathione (GSH), ascorbates (ASC), and carotenoids are categorized as nonenzymatic antioxidant molecules [119,120]. The enhanced levels of ROS, such as hydrogen peroxide (H_2_O_2_), singlet oxygen (^1^O_2_, hydroxyl radicals (OH^−^), and superoxide (O_2_^−^) under salt-stress conditions are quenched or scavenged by these antioxidant enzymes and molecules. The sequential detoxification process begins with the production of SOD in plant cells. SOD acts as the first line of defense and it eliminates superoxide radicals by converting superoxide radicals into oxygen and hydrogen peroxide, thereby reducing hydroxyl radicals. Superoxide radicals reduce metal ions (Fe^3+^ and Cu^2+^), which leads to the formation of hydroxyl radicals that cause severe damage to lipids and cellular membranes due to their oxidating ability.

Hydrogen peroxides and their derivatives generated under salt-stress conditions are then broken down by POX and CAT [121]. Many studies have shown the correlation between salt tolerance and the increased activities of antioxidants. For example, a recent study on *A. tricolor* foliage plants showed an elevated amount of SOD, ascorbate, and APX in a salt-tolerant variety (VA14) to assist ROS detoxification [122]. The production of more malondialdehyde (MDA) under salt-stress conditions is a sign of membrane damage induced by salt stress. Hussain et al. (2022) investigated the salt tolerance of contrasting wheat genotypes and concluded that a lower MDA production in salt-tolerant varieties correlated with a lower-membrane lipid peroxidation [123]. Another study showed that the overexpression of the peroxidase gene *GsPRX9* from wild-type soybean increased salt tolerance with a concomitant enhancement of the antioxidant response [124]. Anthocyanins are a group of antioxidants and their accumulation in plants under salt stress is well documented. Anthocyanin-impaired-response-1 (*air1*) is a mutant gene isolated in Arabidopsis, and it is involved in salt tolerance via regulating various steps of the flavonoid and anthocyanin biosynthesis pathways as the mutant is unable to accumulate anthocyanins under salt stress [125]. These results indicate that many enzymes, molecules, and pigments play protective roles in alleviating oxidative damages towards enhancing plant salt tolerance.

### 3.4. Phytohormone-Mediated Salt Tolerance

Plant hormones or phytohormones are vital endogenous regulatory molecules that regulate plant growth and development. There are nine well-characterized and diverse groups of plant hormones that play sophisticated roles in phytohormone-mediated stress tolerance in plants [126]. Among them, ABA, ethylene, SA, and JA are categorized as stress-responsive hormones, while auxin, GA, cytokinins, brassinosteroids (BRs), and strigolactones (SLs) are considered growth-promotion hormones [59,126]. The phytohormones are intricately interconnected; therefore, stress-response mechanisms are not solely limited to any particular hormone [127]. In this context, the regulation of plant growth adaptation via phytohormone-mediated salt-stress tolerance is briefly discussed.

ABA is an irreplaceable hormone, and it functions as a central integrator to activate an adaptive signaling cascade and regulate gene expression in response to salt stress. Endogenous ABA levels elevate immediately to activate a kinase cascade upon salt-stress exposure [128]. Stomatal closure occurs due to the increased ABA levels to regulate water and osmotic homeostasis. Salt-stress-induced osmotic stress leads to enhanced ABA signaling transduction pathways, which involve a primary component known as sucrose nonfermenting 1-related protein kinases (SnRK2s) [129]. Under salt-stress conditions, the kinase activities of SnRK2.2/2.3/2.6 and the activities of the transcription factors ABA-responsive element (ABRE)-binding protein/ABRE-binding factor (AREB/ABF) further promote stomatal closure [130]. These master transcription factors also regulate the ABRE-mediated transcription and express the downstream target genes for salt tolerance. Additionally, abscisic acid insensitive 1 (ABI1) negatively regulates salt tolerance by inhibiting the kinase activity of SnRK2 and thereby mediates primary root growth [131]. Upon salt-stress exposure, the transcript levels of several ABA biosynthesis genes are upregulated, causing the production of ABA through the methylerythritol 4-phosphate (MEP) pathway. Zeaxanthin oxidase (ZEP), 9-cis-epoxycarotenoid (NCED), and short-chain alcohol dehydrogenase (SCAD) are enzymes induced under salt-stress conditions and they play essential roles in the regulation of the ABA biosynthesis pathway [128,132]. Furthermore, the Ca^2+^ and SOS pathways also coordinate with ABA signaling by preventing SOS2 overactivation [133]. Therefore, ABA employs a complex mechanism in mediating salt-stress response.

Plants take an adaptive mechanism of inhibiting growth to survive harsh salinity environments. Auxin regulates root growth plasticity under salt stress. A reduced polar auxin transport and associated lower auxin accumulation in the roots [134] and the downregulation of auxin-receptor encoding genes (*TRANSPORT INHIBITOR RESPONSE 1* and *AUXIN SIGNALING F-BOX*) [135] cause a lower auxin signaling and therefore downregulate auxin-mediated root growth. Bioactive gibberellin levels are adjusted at different growth stages of plants to enhance salt tolerance through retarded growth. DELLA protein SLR1, an inhibitor of GA signaling [136], and several other GA metabolism-related genes [137] cause reduced GA levels or GA signaling after germination, which is necessary to enhance plant tolerance to salt stress. Cytokinin promotes cell growth, development, and differentiation and is involved in many physiological and biochemical processes in plants. Cytokinin self-sacrifices itself to assist in salt-stress tolerance since it plays opposing roles in the plant adaptation to salt stress. For instance, a loss of the isopentenyl transferase (IPT, a critical enzyme in the cytokinin synthesis pathway) or an overexpression of cytokinin oxidase (CKX, an enzyme that inactivates cytokinin) causes an elevated salt tolerance [138]. As a stress-responsive hormone, ethylene accumulates under salt stress and mediates several critical biological processes. In addition, ethylene signaling also modulates salinity responses. For instance, in a study, the loss of function of the ethylene receptors, ETHYLENE RESPONSE 1 (ETR1) and ETHYLENE INSENSITIVE 4 (EIN4), caused an enhanced salt tolerance. In contrast, the loss of function in the ethylene-positive regulators, EIN2 and EIN3, leads to a hypersensitivity to salt stress [139]. Therefore, phytohormones and their sophisticated crosstalks are vital to salinity stress signaling, and they maintain a balance between plant growth and stress responses.

### 3.5. Epigenetic Regulations on Salt-Stress Tolerance

Over the last decade, transcriptional responses have been broadly studied to identify the distinct signaling cascades involved in salt-stress signaling, leading to the identification of various regulatory proteins and their targets. Epigenetics is the regulation of gene expression through heritable covalent alterations in chromatin architecture to facilitate the accessibility of transcriptional machinery. DNA methylation, histone modifications, histone variants, and some noncoding RNAs (ncRNA) are the epigenetic components involved in critical biological processes, including the expression of genes and genome stability [140].

DNA methylation is a widely investigated epigenetic modification, which includes the insertion of a methyl group at the 5’ position of cytosine in DNA sequence contexts such as CG, CHG, and CHH (H represents A, T, or C). A perturbation in the methylation patterns and its consequences on gene expression under saline conditions have been extensively demonstrated. For example, the methylation level at the promoter of a salt-stress-responsive gene, the flavonol synthase gene *TaFLS1* in wheat, was notably lower in a salt-tolerant cultivar, suggesting a potential role of DNA methylation in salt tolerance [141]. In addition to DNA methylation, histone tails or the N’ termini of histone proteins are covalently modified by acetylation, methylation, phosphorylation, sumoylation, and ubiquitination. Specific histone modification enzymes determine these modifications and are imperative in gene regulation under salt stress. Feng et al. (2022) recently demonstrated the role of GsMYST1, a protein phylogenetically homologous to histone acetyltransferase in wild soybean. They showed that the coordinated phosphorylation of GsMYST1 by the GsSnRK1 kinase and the consequent acetylation of the histone H4 on the target genes upregulated stress-responsive gene expression, leading to an increased salt tolerance [142]. Histone methylation is a dynamic process that elevates histones’ hydrophobicity and creates novel binding sites. The methylation at H3K4me3 and H3K27me3 in castor beans regulates the transcription of a critical salinity response regulator gene, *RSM1* (encoding the RADIALIS LIKE SANT-an MYB related transcription factor), which is involved in the ABA-mediated salt-stress signaling [143].

Besides the abovementioned factors influencing chromatin dynamics, chromatin remodeling also changes gene expression. The disruption of histone–DNA interactions alters the accessibility of the transcription machinery to the specific DNA region [144]. Chromatin remodeling factor (CHR), which encompasses various subfamilies of ATPases, such as SWI/SNF, the imitation switch (ISWI), and chromodomain and helicase-like domain (CHD), can play a role in both ATP-dependent chromatin remodeling and post-translational histone modifications [145]. According to a study by Nguyen et al. (2019), the *brm-3* mutant showed increased transcript levels of ABA-related *PP2C* genes (*ABI1*, *ABI2*, and *HAI1*) when exposed to NaCl treatment [146]. This suggests that BRM, a chromatin-remodeling ATPase known as BRAHMA, might function as a suppressor of these genes. These researchers found that the chromatin associated with the *PP2C* genes changed from a suppression state (by a repressor) to a transcription state (by an activator) in response to salt stress.

Recent research on epigenetic modifications suggests that gaining a better understanding of these processes and utilizing genome-editing technologies can potentially lead to crop improvement under salt-stress conditions. As our knowledge of epigenetics continues to advance, it will be possible to develop alternative strategies for crop breeding that can enhance salt tolerance and ultimately increase crop yields.

## 4. Crop Breeding Strategies for Achieving Salt Tolerance

Numerous studies have explored the screening and breeding of crop plants to enhance salinity tolerance, and the subject has been periodically reviewed previously [147,148,149]. Advances in identifying genetic markers, molecular markers, germplasm modification, and mapping have improved salt tolerance. Conventional breeding methods such as hybridization, selection, polyploidy, and introgression are considerably effective in enhancing salt tolerance, and the utilization of wild relatives of crop plants as a source of salt-tolerant genes to increase the range of variation for salt tolerance improvement is still ongoing. However, conventional breeding faces a serious challenge due to the limited genetic variation present in the gene pool of most crop species. Other reasons contribute to the limited success of conventional breeding, including the labor-intensive nature of the process, the transfer of undesirable genes along with desirable traits, unpredictable outcomes, the inability to introduce non-native traits, and reproductive barriers all restrict the transfer of favorable alleles from interspecific and intergeneric sources [150]. Conventional breeding’s limited success in enhancing plant stress tolerance can also be attributed to breeders’ inclination to test genetic materials under ideal conditions. Additionally, the intricate nature of abiotic stresses and plants’ varying sensitivity to these stresses at different developmental stages further complicate the selection criteria for increased salt-stress tolerance [151]. In addition to conventional breeding, breeding strategies such as mutational breeding [152], double haploid production [153], and marker-assisted breeding [154] seem to be more attractive alternatives that could contribute to the development of salt-tolerant crops.

## 5. Utilization of QTL Knowledge on Salt Tolerance

Quantitative trait loci (QTLs) and marker-assisted selection can provide many benefits compared to direct phenotypic screening. Using PCR-based techniques to identify the genetic markers can considerably reduce the time and environmental impacts required to screen genotypes. It has been substantiated that salt tolerance is a multifaceted trait, and QTLs genetically control the inheritance of salt-tolerance traits and have both additive and dominant effects [155,156].

A plethora of studies have been conducted thus far on QTL analyses to identify QTLs linked to salt tolerance in various crops. For instance, in rice, multiple QTLs have been associated with salinity tolerance during the reproductive phase, with the “Saltol” QTL demonstrating a strong association with salinity tolerance [157]. “Saltol” is the most extensively researched QTL, responsible for a high K^+^/Na^+^ ratio and low Na^+^ uptake under salinity stress. Furthermore, in a study conducted on a rice backcross inbred line population, 23 loci were identified for germination parameters at the germination stage. In comparison, 46 loci were identified for various morphological and physiological parameters at the seedling stage. This population was developed by crossing an Africa rice (ACC9) with an Indica cultivar (ZS97) [158]. Another study created a genetic linkage map employing 532 molecular markers spanning 1341.1 cM to identify the loci associated with salt tolerance in *Brassica napus*. A candidate gene in this region, *Bra003640*, was identified as being linked to salt tolerance [159].

The progress in molecular biology has paved the way for the emergence of DNA markers that facilitate the identification of QTLs. In the past two decades, we have witnessed noteworthy advancements in molecular marker technology, enabling the creation of comprehensive molecular linkage maps. Consequently, there has been substantial headway in marker-assisted selection procedures, which could eventually facilitate the combination of favorable trait identification and significantly enhance crop salt tolerance.

## 6. Genetic Engineering of Salt Tolerance in Plants

Using recombinant DNA techniques and transcriptomic and genomic technologies, a large number of genes that are either upregulated or downregulated in response to salinity stress have been discovered and characterized. Some of these differentially regulated genes encode proteins that play vital roles in stress-related growth and metabolism. In contrast, other genes encode regulatory proteins, such as salt-responsive transcription factors capable of controlling the expression of many target genes by binding to the specific *cis*-elements in their promoters [119]. Numerous studies in diverse plants species have demonstrated the functions of salt-stress-responsive transcription factors including ERF/AP2, bZIP, WRKY, NAC, MYB, and C2H2 zinc finger proteins (C2H2-ZFP) in plant salt tolerance. Evidence has shown that common regulatory mechanisms operate in different species and genotypes of plants. For instance, differential expression studies on four different genotypes *of Brassica* showed an upregulation of genes that encode components of the SOS pathway such as *SOS1* (plasma membrane Na^+^/K^+^ antiporter), *SOS2* (protein kinase), *SOS3* (calcium-binding protein), and *NHX1* (vacuolar Na^+^/K^+^ antiporter) in response to salinity stress [160]. Various rice genotypes were investigated for a differential expression analysis under salt stress, and the results showed a higher expression of *OsHKT*, *OsNHX*, and *OsSOS1*, which are all related to Na^+^/K^+^ homeostasis [161].

Among the various mechanisms involved in salt tolerance, targeting suitable candidate genes that control water movement through ion homeostasis, uptake and transport, compartmentalization, and utilization of aquaporin channels is an effective strategy for developing salt-tolerant plants [162]. Engineering plants by overexpressing or co-overexpressing beneficial genes encoding for transporters or antiporters in the root tissues to mitigate excessive ion uptake and transport proves to be a useful strategy. A list of genetic modifications of ion transport components in plants for enhanced salt tolerance is shown in Table 1. This list includes the overexpression of proton pumps (plasma membrane H^+^-ATPase and vacuolar H^+^-pyrophosphatases), Na^+^/H^+^ antiporters, and potassium transporters. Nonetheless, complex interactions, regulatory networks, and competitive uptake systems of essential ions under salt stress must be studied in field conditions, not just laboratory conditions.

In the past few decades, transgenic approaches have revolved around manipulating genes in various signaling and metabolic pathways, including ion homeostasis, compartmentalization, osmoprotectant accumulation, ROS scavenging, and transcriptional regulations. These successful strategies have enabled scientists to genetically engineer salt tolerance in transgenic plants precisely and predictably. However, single gene manipulation would not be an ideal strategy for achieving high salt tolerance as numerous experiments have indicated that the overexpression of single genes could only increase salt tolerance to around 100 mM to 150 mM NaCl as evidenced by transgenic plants overexpressing *AtNHX1* [112,113,114,115,116], *SOS1* [86,87], and *AVP1* [103,104,106,109,110]. To further increase salt tolerance, an approach using the co-overexpression of two genes that function synergically was taken by many scientists, and the salt tolerance was increased to 200 mM to 250 mM NaCl as demonstrated by transgenic plants co-overexpressing *AVP1* and *SOS1* [85], *AVP1* and *OsSIZ1* [101], *AVP1* and *PP2A-C5* [102], and *AVP1* and *AtNHX1* [105]. Recently, we showed that it was possible to further increase the salt tolerance to 300 mM NaCl by co-overexpressing three genes, *AVP1*, *AtCLCc*, and *PP2A-C5*, in transgenic Arabidopsis plants [99], indicating that co-overexpressing several genes that are relevant to salt tolerance was indeed an effective approach in achieving a high salt tolerance.

Apart from the abovementioned strategies, recent advancements in new breeding techniques, such as genome editing, have been used to engineer desired genes in model plants and crop species [163]. Clustered regularly interspaced short palindromic repeats (CRISPR)/CRISPR-associated protein (CRISPR/Cas) is one of the newest additions to genome editing tools, which targets structural genes, regulatory genes, and *cis*-regulatory elements associated with salt-response pathways [164]. For instance, the *SOS1* locus in rice was edited by inserting a 60 bp translational enhancer at 35.7% insertion frequency to achieve a higher salt tolerance [165]. Knocking out a barley gene (i.e., *HVP10*) through the CRISPR/Cas9 mediated editing led to the inhibition of plant growth and excessive Na^+^ concentration in shoots [166,167]. Additionally, genome editing by using customized nucleases (zinc-finger nucleases (ZFNs), transcriptional activator-like effector nucleases (TALENs)) to target mutations allows precise and predictable modifications to be utilized in crop improvement. Small-RNA-mediated (micro-RNAs and small interfering RNAs) gene silencing and RNA interference (RNAi) technology has become a widely accepted method that enables a precise and multiplex editing of target genes without affecting the expression of other genes. RNAi can function as a master regulator by suppressing the transcription or mediating post-transcriptional gene silencing, thereby playing a vital role in combating biotic and abiotic stresses [168,169,170]. The development of computational tools and databases ease the identification of stress-responsive miRNAs and their targets regarding improved salt tolerance. For example, the role of *miR172a* in soybean was reported to enhance salt tolerance and facilitate long-distance stress signaling as the engineered plants survived and grew better than control plants under severe salt-stress conditions [171]. The above studies demonstrate the manipulation of candidate genes through genome editing and RNAi technology, which could lead to an increased salt tolerance. Generating transgenic crops through genetic engineering is still challenging, requiring further research and field evaluation. Therefore, it is essential to understand the complex nature of salt tolerance in plants, the regulatory networks of salt signaling, and species-specific differences in salt tolerance in plants.

**Table 1:** List of ion transporter genes genetically engineered to enhance salt tolerance.

Source Organism	Gene	Transgenic Host	Improved Trait under Salinity Stress	References
**Vacuolar Na^+^(K^+^)/H^+^ antiporter**				
*Arabidopsis thaliana*	*AtNHX1*	*Actinidia deliciosa*	Greater osmotic adjustment and antioxidant capacity in transgenics	[172]
*Arabidopsis thaliana*	*AtNHX1*	*Fagopyrum esculentum*	Accumulation of more rutin	[173]
*Arabidopsis thaliana*	*AtNHX2*	*Gossypium hirsutum* L.	Greater yield of better-quality cotton fiber	[115]
*Arabidopsis thaliana*	*AtNHX3*	*Brassica napus*	Unaffected seed yield and seed oil quality under saline conditions	[174]
*Arabidopsis thaliana*	*AtNHX4*	*Arachis hypogaea* L.	Elevated rate of photosynthesis	[175]
*Arabidopsis thaliana*	*AtNHX3*	*Beta vulgaris*	Increased salt accumulation in leaves, greater root storage with higher soluble sugars	[176]
*Gossypium hirsutum*	*GhNHX1*	*Nicotiana tabacum*	Increased Na^+^ compartmentalization	[177]
*Pennisetum glaucum*	*PgNHX1*	*Oryza sativa*	Robust root system	[178]
*Solanum torvum*	*StNHX1*	*Glycine max*	Leaves appearance with lower scorch scores and a lower content of Na^+^ and malondialdehyde	[179]
**Plasma membrane Na^+^/H^+^ antiporter system**				
*Arabidopsis thaliana* L. (wild type)	*AtSOS1*	*Arabidopsis thaliana*	Better root growth, increased germination rate, elevated chlorophyll content, and reduced accumulation of Na^+^	[180]
*Arabidopsis thaliana*	*AtSOS1*	*Arabidopsis thaliana*	Better growth and higher survival rate	[86]
*Arabidopsis thaliana*	*AtSOS2*	*Nicotiana tabacum cv. Xanthi-nc*	Superior growth and increased germination rate	[87]
*Gossypium hirsutum*	*GhSOS1*	*Arabidopsis thaliana*	Lower MDA content and decreased Na^+^/K^+^ ratio	[181]
**Plasma membrane-bound high-affinity potassium transporters**				
*Arabidopsis thaliana*	*AtHKT1*	*Solanum tuberosum* L.	Alleviation of salt-induced damages in potato	[182]
*Populus trichocarpa*	*PeHKT1;1*	*Populus davidiana × Populus bolleana*	Better relative growth rate, higher catalase (CAT), peroxidase (POD), and superoxide dismutase (SOD)	[183]
*Glycine max*	*GmHKT1;4*	*Nicotiana tabacum*	Greater amount of K^+^ and less Na^+^, maintaining a lower Na^+^/K^+^ ratio in roots under alkaline and saline conditions	[184]
**Vacuolar H^+^-pyrophosphatase**				
*Arabidopsis thaliana*	*AVP1*	*Arabidopsis thaliana*	Increased sequestration of solutes into vacuole	[103]
*Arabidopsis thaliana*	*AVP1*	*Arachis hypogaea*	Greater biomass and elevated photosynthetic rate	[109]
*Arabidopsis thaliana*	*AVP1*	*Gossypium hirsutum*	Improved salt tolerance and greater fiber yield under greenhouse and field conditions	[106,107]
*Arabidopsis thaliana*	*AVP1*	*Hordeum vulgare*	Larger biomass and greater grain yield	[110]
**Co-overexpression of genes**				
*Arabidopsis thaliana*	*AtNHX1* and *SOS1*	*Arabidopsis thaliana*	Salt tolerance up to 250 mM of NaCl	[85]
*Arabidopsis thaliana*	*AtNHX1* and *Bar* gene	*Vigna radiata* L. Wilczek	Transgenic plants with better ion homeostasis and reduced oxidative stress	[185]
*Arabidopsis thaliana*	*AtNHX1* and *AVP1*	*Gossypium hirsutum*	Robust growth with a larger root system and greater fiber yield	[105]
*Arabidopsis thaliana* and *Oryza sativa*	*AVP1* and *OsSIZ1* (SUMO E3 Ligase)	*Arabidopsis thaliana* (Overexpression)	Increased abiotic stress tolerance including salt stress	[101]
*Arabidopsis thaliana* and *Larrea tridentata*	*AVP1* and Rubisco activase gene *RCA*	*Arabidopsis thaliana* (Overexpression)	Higher biomass and seed yield	[100]
*Arabidopsis thaliana*	Vacuolar H^+^-pyrophosphatase, catalytic subunit of protein phosphatase 2A, and chloride ion channel protein (*AVP1*, *PP2A-C5* and *AtCLCc*)	*Arabidopsis thaliana* (Overexpression)	Robust growth with a greater number of viable seeds	[99]
*Arabidopsis thaliana*	Vacuolar H^+^-pyrophosphatase and catalytic subunit of protein phosphatase 2A (*AVP1* and *PP2A-C5*)	*Arabidopsis thaliana* (Overexpression)	Enhanced salt tolerance to NaCl, KNO_3_, and LiCl	[102]
**Other proton pumps**				
*Spartina alterniflora*	Vacuolar H^+^-ATPase (*saVHAc1*)	*Oryza sativa*	Enhanced yield observed under salinity conditions	[186]
*Sesuvium portulacastrum*	Plasma membrane H^+^-ATPase (*SpAHA1*)	*Arabidopsis thaliana*	Robust growth with longer roots, greater biomass, and a higher rate of seed germination	[187]

## 7. Concluding Remarks and Future Perspectives

Over the last decade, significant progress has been made in understanding plant salt-stress responses and salt-tolerance mechanisms. Plant salt tolerance is achieved via a complex interaction at the genetic, cellular, and physiological levels. A large body of evidence has suggested several salt-tolerance mechanisms, such as maintaining ion homeostasis, regulating ion transport, osmotic regulation, increasing antioxidant metabolism, and transgenic approaches have successfully utilized these mechanisms in generating salt-tolerant plants. Improving salt-stress tolerance in crops has become more feasible with the successful application of gene editing technologies and computational tools. Nonetheless, there are still challenges ahead. The integration of information from genomic, transcriptomic, proteomic, and metabolomics research needs to be improved, and a comprehensive approach is required to discover the major pathways that determine salinity tolerance. The current knowledge of transmembrane ion transport, sensor, and receptor in signaling transduction and long-distance signaling is still lacking. The research on intercellular and intracellular molecular interactions involved in salt-stress response should be prioritized in the future. Unfortunately, our knowledge of ion transporters involved in salt intake, exclusion, sequestration, and transport is still limited for most crops. The molecular processes driving root-to-shoot interactions must be more thoroughly understood. Future research should focus on the crosstalk between various hormones in response to salt stress. Improving salt tolerance is thus a complicated and challenging task, particularly if the goal is to generate crop plants that are both salt-tolerant and agronomically important.

## Data Availability

Not applicable.
